# mRNA profiles of cytokine receptors in unstimulated peripheral blood mononuclear cells from patients with chronic idiopathic urticaria

**DOI:** 10.1016/S1674-8301(11)60018-3

**Published:** 2011-03

**Authors:** Jianming Gao, Aizhen Yang, Min Chen, Ansheng Li, Xu Yao, Yumei Li, Shihai Xie, Xueyuan Yang, Liansheng Zhong, Zhiqiang Chen

**Affiliations:** a Department of Dermatology, Haidian Hospital, Beijing 10080, China; b Oncology Center of Chinese PLA 81^st^ Hospital, Nanjing 210002, Jiangsu, China; c Section of Clinical Immunology, Institute of Dermatology, Chinese Academy of Medical Sciences & Peking Union Medical College, Nanjing 210042, Jiangsu, China

**Keywords:** urticaria, cytokine, chemokine, receptor

## Abstract

This present study was aimed to investigate the roles of the receptors of Th1/Th2 cytokines and chemokines in the pathogenesis of chronic idiopathic urticaria (CIU). Thirty patients with CIU, 30 patients with dermographism and 30 healthy controls were randomly enrolled. Reverse transcription-PCR (RT-PCR) was used to analyze the mRNA of cytokine receptors in peripheral blood mononuclear cells (PBMCs). The mRNA levels of tumor necrosis factor receptor (*TNFR*), interferon-γ receptor (*IFN*-*γR*), and interleukin-10 receptor (*IL*-*10R*) were statistically increased in the CIU group (*P* < 0.05), while *IL*-*2R, IL*-*4R, IL*-*6R*, and *IL*-*13R* showed no significant differences between the CIU and other groups. The mRNA levels of *CCR3* and *CCR6* were statistically increased in the CIU group (*P* < 0.05). The toll-like receptor 2 (*TLR2*) mRNA level was significantly lower in the CIU group than the healthy control group (*P* < 0.05). These findings indicate that the regulation of mRNA of *TNFR, IFN*-*γR, IL*-*10R*, *CCR3, CCR6* and *TLR2* may be involved in the pathogenesis of CIU.

## INTRODUCTION

Chronic idiopathic urticaria (CIU) is a chronic inflammatory skin disease characterized by short-lived, pruritous swellings of the skin, mouth, and genitalia due to transient leakage of plasma from small blood vessels into the surrounding connective tissue. As patients often experience itching, the physical and mental health of the patients with CIU is greatly influenced. The etiology and pathogenesis of CIU are complicated and still remain to be elucidated[1]. Some of the cases were found to have autoantibody against the thyroid gland[2]. The histopathological lesions of the skin in CIU is usually marked by dermal edema, and perivascular mononuclear cell infiltration predominantly with lymphocytes, eosinophils and mast cells. The actions of these infiltrating lymphocytes in the pathogenesis of CIU as well as the linkage between the infiltrating lymphocytes and other inflammatory cells in CIU are not fully delineated so far. Although there have been numerous studies on inflammatory mediators from other cells such as eosinophils and mast cells, the roles of lymphocytes in CIU are still under investigation. On the other hand, the main therapeutic strategy for CIU is against the action of histamine and other inflammatory mediators such as cysteinyl leukotrienes, which are secreted by mast cells, basophils or eosinophils. Autoimmune urticaria, a heterogeneous subset of CIU, is related to an antibody that belongs to the IgG isotype and reacts against the α-chain of the high affinity IgE receptor (FcϵRI) of basophils and mast cells, or alternatively against IgE itself [3]. Functional properties of antibodies can be examined *in vivo* by intradermal injection of autologous serum (autologous serum skin test, ASST), which can induce a wheal-and-flare response in patients with CIU. Further evidence could be seen *in vitro* with the release of histamine from basophils and mast cells elicited by the addition of CIU patient serum. Apart from the factors of humoral immunity such as autoantibody, there may be some cellular immunity factors involved in the pathogenesis of CIU, although there are few literatures about it. One of the investigations is on the cytokine expression profiles in CIU[4]. In the present study, we examined the expression profiles of cytokine receptors in the PBMCs of CIU patients to investigate the possible actions of the receptors of Th1/Th2 cytokines and chemokines in the pathogenesis of CIU.

## MATERIALS AND METHODS

### Subjects

Thirty patients (12 males and 18 females; age ranged from 18 to 65 years) with CIU were randomly enrolled from the out-patient-department of CAMS Hospital for skin diseases. The average disease course of these patients was 40.6 months (2 months to 14 years). Meanwhile, thirty patients (13 males and 17 females, age ranged from 19 to 59 years) with dermographism were randomly enrolled, with an average disease course of 39.6 months (3 months to 21 years). Additionally, thirty healthy volunteers (14 males and 16 females, with age of 22-54 years) were enrolled in this study as healthy controls (HCs), who themselves and their immediate family members had no history of allergy. All patients and control subjects signed informed consent form.

### Inclusion and exclusion criteria of CIU

The inclusion criteria of CIU were: 1) The patient was over 18 years old. 2) The wheals were seen when enrolled in the study, and the patient had a history of recurrent wheals over 6 weeks with frequencies of 4 times a week or more. 3) The allergy to food or drug was ruled out and there was no definite cause clinically. 4) The patient had no history of allergic diseases such as rhinitis, asthma or atopic dermatitis, no history of autoimmune diseases or parasite infection. 5) Serum specific anti-IgE antibodies were negative as detected by Allergy Screen assay (MEDIWISS Analytic, Moers). 6) Serological examination for hepatitis B and C showed negative results. 7) Serum test for anti-*Helicobacter pylori* antibody, antithyroid autoantibodies and antinuclear autoantibodies showed negative results.

The exclusion criteria of CIU were: 1) the wheals lasted over 24 h; 2) patients with other types of urticaria such as physical urticaria, hereditary angioedma, drug-induced urticaria or urticarial vasculitis. 3) pregnant woman or lactating woman; 4) the patients had taken corticosteroids or immunomodulants during the past 4 weeks or taken antihistamines during the past 3 days; 5) patients with concomitant allergic contact dermatitis, atopic dermatitis, eczema or other pruritic skin diseases; 6) patients with abnormal test results of blood hematology tests, blood chemistry tests, urine analysis or stool analysis.

### Peripheral blood mononuclear cell (PBMCs) isolation

Venous blood of 6 mL from CIU patients and control subjects was collected, anti-agglutinated with ethylenediaminetetraacetic acid (EDTA) and diluted with an equal volume of cold Hanks solution. PBMCs were obtained by Ficoll density gradient centrifugation with lymphocyte separation medium (Tian Jing TDB, China).

### RNA extraction

Total RNA was extracted from PBMCs (2×10^7^ cells) of 30 CIU patients, 30 dermographism patients and 30 healthy individuals by the guanidinium isothiocyanate/phenol extraction method (TRIzol, Gibco BRL, Germany). RNA was quantified using absorbance at 260 nm. The purity was checked by reading absorbance at 260/280 nm, and the integrity was detected by agarose-formaldehyde gel electrophoresis after staining with ethidium bromide.

### RT-PCR

Reverse transcription of 1 µg of total RNA was performed by using the ‘Reverse Transcription System’ (Promega Corporation, Madison, WI, USA) according to the manufacturer's protocol. cDNA (12 µL) was subsequently used as a template in PCR. Amplication was performed in a DNA thermal cycler (2400GeneAmp PCR System, Perkin Elmer, USA) as follows: initial denaturation at 94°C for 5 min, 35 cycles of amplification (94°C for 60 s, 60°C for 60 s, and 72°C for 60 s) followed by final extension at 72°C for 7 min. The sequences of primers used for PCR are listed in ***table 1***.

**Table 1:** Primer sequences for PCR reactions

Genes	Primer sets	Sequences	Size(bp)
β-actin	sense	5′-CAACTCCATCATGAAGTGTA-3′	180
	antisense	5′-CCACACGGAGTACGCGCTG-3′	
IFN-γR	sense	5′-GAGCCAGCGACCGTCGGTAGC-3′	335
	antisense	5′-TTCTTTTTGTCCAACCCTGGC-3′	
IL-2R	sense	5′-AAATCAAAGGTGCTAAAT-3′	362
	antisense	5′- TGAACTGGGAAGTTGGAA-3′	
IL-4R	sense	5′-CCCCCACCAGTGGCTATC-3′	162
	antisense	5′-GCCCCAAACCCACATTTC-3′	
IL-6R	sense	5′-CAAGCCTCCCAGTGCAAGAT-3′	309
	antisense	5′- ATTGCTGATGTCATAAGGGC-3′	
IL-10R	sense	5′-GTACCACAGCAATGGCTACC-3′	672
	antisense	5′- TGCAGGTCCAAGTTCTTCAGC-3′	
IL-13R	sense	5′- GCTCCGGAAACTCGTCGTTC-3′	676
	antisense	5′-GGAAGAACACCAGGGACCAT-3′	
TNFR	sense	5′- CAAGAGCCTGAGTAGGTGGTTTG-3′	185
	antisense	5′- CTGCTTATGCACTATGAAAAAGG-3′	
CCR3	sense	5′- TGGCGGTGTTTTTCATTTTC-3′	315
	antisense	5′- CCGGCTCTGCTGTGGAT-3′	
CCR6	sense	5′- GAGCCCATCAGGAAGCTGCTG-3′	316
	antisense	5′-GGCAGCAGTGCAGGAAAGCCAGGAC -3′	
CXCR3	sense	5′- TCCTTGAGGTGAGTGACCACAAA-3′	584
	antisense	5′-CTCGTCGTGGTGGGCCGACAG-3′	
CX3CR1	sense	5′-GTAGTGTTTGCCCTCACCAACA-3′	502
	antisense	5′-ACAGCGTCTGGATGATTCTGAA-3′	
CysLTR-1	sense	5′-ATGACAGCCATGAGCTTTTTC-3′	480
	antisense	5′-CATTCTAAGGACAGAATCACA-3′′	
TLR2	sense	5′- TCGGAATGTCACAGGACAGC-3′	368
	antisense	5′- CAGTTCATACTTGCACCACTCAC-3′	
TLR5	sense	5′-CCTCATGACCATCCTCACAGTCAC-3′	355
	antisense	5′-GGCTTCAAGGCACCAGCCATCTC-3′	

### Statistical analysis

The PCR products were seperated by electrophoresis on a 2% agarose gel in the presence of ethidium bromide stain. All images were visualized under ultraviolet (UV) illumination (Gel Doc 2000 System, New England Biolab, Inc. Beverly, MA, USA). The data were analyzed by Quantity One 1D Analysis Software (Gel Doc 2000 System, New England Biolab, Inc., Beverly, MA, USA). All data were expressed as mean±SE, and the differences between CIU patients, dermographism patients and healthy controls were analyzed by the *t*-test. To determine the statistical significance of each comparison, we performed random permutation analysis to define the *P*-value thresholds. Differences were considered significant if *P* values were 0.05 or less. All data were analyzed using SPSS11.5 (SPSS, Inc., Chicago, IL, USA) for Windows.

## RESULTS

### Receptors of Th1/Th2 cytokines

**Fig. 1:**
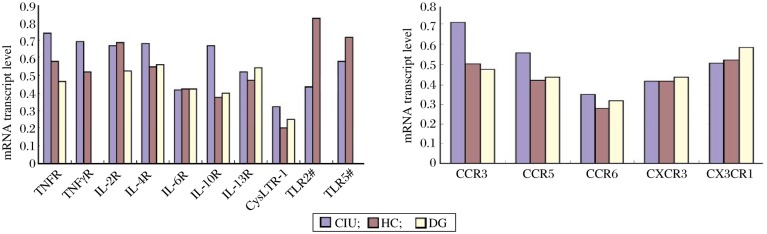
Profiles of the mRNA transcripts of cytokines (A) and chemokines (B) in patients with CIU or DG and HC. The levels of mRNA were examined by RT-PCR. The detection of *IFNR*, *TLR2* and *TLR5* mRNA was not conducted in the DG group in A. CIU: chronic idiopathic urticaria; DG: dermographism; HG: healthy control.

The mRNA expression of Th1/Th2 cytokine receptors in unstimulated PBMCs was detected in all the subjects. The mean levels of *INFγR*, *TNFR* and *IL*-*10R* mRNA in CIU patients were significantly higher than those in healthy controls (*P* < 0.05, all) (***Table 2*** and ***Fig. 1***). On the other hand, there was no statistical difference in the mRNA transcript levels of *IL*-*2R, IL*-*4R, IL*-*6R*, and *IL*-*13R* between CIU patients and heathy controls. The mean mRNA transcript levels of *TNFR* and *IL*-*10R* in CIU patients were significantly higher than those of patients with dermographism (*P* < 0.05, both). Furthermore, no statistical differences were noted in the mRNA levels of all the cytokine receptors examined between the dermographism group and healthy control group (***Table 2***).

**Table 2:** Mean mRNA transcript levels of Th1/Th2 cytokine receptors and TLRs in the study subjects

CR	CIU	HC	DG	*P1*	*P2*	*P3*
*TNFR*	0.737±0.220	0.580±0.367	0.466±0.179	0.048	0.000	0.135
*IFNγR*	0.691±0.153	0.518±0.169	ND	0.000	—	—
*IL-2R*	0.670±0.527	0.688±0.357	0.526±0.334	0.872	0.213	0.074
*IL-4R*	0.679±0.300	0.549±0.267	0.561±0.157	0.082	0.060	0.841
*IL-6R*	0.415±0.242	0.425±0.310	0.422±0.154	0.892	0.896	0.964
*IL-10R*	0.670±0.280	0.373±0.150	0.400±0.125	0.000	0.000	0.447
*IL-13R*	0.518±0.370	0.469±0.410	0.541±0.275	0.631	0.789	0.432
*CysLTR-1*	0.321±0.340	0.202±0.116	0.251±0.106	0.073	0.285	0.088
*TLR2*^#^	0.436±0.243	0.821±0.339	ND	0.000	**—**	**—**
*TLR5*^#^	0.580±0.285	0.716±0.350	ND	0.164	**—**	**—**

*P1* compared between the CIU group and the HC group; *P2* compared between the CIU group and the DG group; *P3* compared between the DG group and the HC group. ^#^ The detection was conducted in 30 healthy controls and 19 CIU cases. CIU: chronic idiopathic urticaria; CR: cytokine receptors; HC: healthy controls; DG: dermographism; ND: not detected.

(mean±SE)

### Receptors of chemokines

The mRNA expression of chemokine receptors in unstimulated PBMCs was detected in all the subjects. The mean levels of *CCR3* and *CCR6* mRNA transcripts were statistically increased in the CIU group (*P* < 0.05), compared with those in the healthy control group. On the other hand, there were no statistical differences between CIU patients and normal controls in the mean levels of *CCR5, CX3CR1* and *CXCR3* mRNA transcripts. Apart from *CCR3*, the mRNA levels of all the other chemokine receptors examined showed no significant differences between the CIU group and dermographism group (***Table 3*** and ***Fig. 2***).

**Table 3:** Mean mRNA transcript levels of chemokine receptors in the study subjects

CR	CIU	HC	DG	*PI*	*P2*	*P3*
*CCR3*	0.715±0.213	0.505±0.261	0.479±0.187	0.001	0.000	0.653
*CCR5*	0.563±0.513	0.422±0.192	0.438±0.190	0.164	0.214	0.754
*CCR6*	0.350±0.158	0.277±0.118	0.318±0.097	0.047	0.340	0.152
*CXCR3*	0.416±0.208	0.419±0.209	0.438±0.188	0.953	0.667	0.713
*CX3CR1*	0.510±0.258	0.527±0.135	0.588±0.242	0.741	0.667	0.233

*P1* compared between the CIU group and the HC group; *P2* compared between the CIU group and the DG group; *P3* compared between the DG group and the HC group. CIU: chronic idiopathic urticaria; CR: cytokine receptors; HC: healthy controls; DG: dermographism.

(mean±SE)

### Receptors of CysLTR-1

There was no significant difference among the three groups in the mean levels of *CysLTR*-*1* mRNA transcripts as shown in ***Table 2***.

### Toll-like receptors

The mRNA expression of toll-like receptors (*TLR*) in unstimulated PBMCs was detected in 30 healthy controls and 19 cases of CIU patients. The mean level of *TLR2* mRNA transcripts in the CIU group (0.436±0.242) was significantly lower than that of healthy controls (0.821±0.339) (*P* < 0.05). There was no significant difference in *TLR5* mRNA expression levels between the two groups (***Table 2***).

## DISCUSSION

Urticaria is one of the most common causes of consultation in dermatology, allergology and emergency care. The patients with urticaria usually suffer important alterations in the quality of life, although most of them have no systemic manifestations. The roles of lymphocytes in the pathogenesis of CIU are not as well documented as those of mast cells, eosinophils and basophils. The histopathological features of chronic urticaria are characterized by a perivascular infiltrate around the venules without vasculitis or immune complex deposits at the expense of CD4^+^ cells with mixed Th1/Th2 characteristics and monocytes, but no B lymphocytes, and a variable presence of granulocytes (polymorphonuclear cells, eosinophils, basophils) that form a late-phase infiltrate. Diminished peripheral basophil counts may be observed along with eosinophil activation products (MBP and ECP), and the presence of adhesion molecules (integrins and selectins) reflecting the presence of endothelial cell activation. Meanwhile, there seems to be no report on a unique histological manifestation of CIU different from other types of urticaria so far. Therefore, it is easily understood why the exact etiology of CIU is still unknown till now. CIU cases are frequently seen in patients with autoimmune disorders (e.g., thyroiditis) with more severe clinical urticarial features than other CU patients. These phenomenon associated with autoimmunity suggest a possible role of lymphocytes in the etiology of CIU[5],[6]. However, the detected antoantibodies or concomitant autoimmune diseases (such as thyroiditis) are only seen in no more than one third cases of CIU, which hint a possibility that an alternative mechanism other than humeral factors (e.g. antibody) may play some roles in the immunopathogenesis of CIU[7],[8]. The present study was conducted to explore the roles of cellular immunity in the pathogenesis of CIU, i.e. the actions of cytokines and chemokines in CIU.

It seems that an early event in the immunopathology of CIU is the activation of mast cells followed by lymphocyte-mediated hypersensitivity reactions with a non-polarized cytokine profile (Th0 or alternatively a mixed Th1/Th2 type profile)[9]–[11]. This kind of profile may be due to concomitant granulocyte cell-mediated hypersensitivity reactions[12]–[15]. It is necessary to explore the details of lymphocyte-mediated hypersensitivity reactions in CIU. Grattan *et al*.[16] found that mast cell degranulation occurred in early lesions in the autologous serum-induced wheals of CIU patients and T lymphocytes, perivascular neutrophils and eosinophils increased from 30 min to 2 h. At 24 h, CD4^+^ cells significantly outnumbered other types of cells and at 48 h, the neutrophils were clearing, but eosinophils and lymphocytes persisted. Similar findings were reported by other authors[17]–[20]. Except for CD4^+^ T cells, CD8^+^ T cells and CD4/CD8 ratio, the proinflammatory cytokines[21]–[23] or chemokines[24],[25] in CIU were studied. These cytokines are involved in the actions of lymphocytes as well as granulocytes.

A method to distinguish lymphocyte-mediated hypersensitivity from granulocyte cell-mediated hypersensitivity is to detect the receptor profiles of cytokine on lymphocytes. According to previous studies[26], a polarized cytokine profile (i.e.Th1- or Th2-prominent) does not seem to exist, which is another reason that we investigated the receptor profile of cytokines in CIU. The present study revealed that there is neither a Th1-prominent nor a Th2-prominent profile of cytokine receptors in CIU patients. In this study, we found that there were elevated mRNA levels of *TNFR, IFN*-*γR*, and *IL*-*10R. TNF* and *TNFR*-related superfamily proteins play central roles. The TNF/TNFR superfamily proteins coordinate the social context of cells in the adaptive immunity that enables lymphocytes to maximally respond to antigen-directed immunity[27]. TNFR signaling plays an important role not only in host defense but also in T-cell dependent inflammation. Furthermore, TNFR could act as mediators in autoimmune diseases[28]. The elevated mRNA expression levels of TNFR in CIU patients in this study probably reflect its actions in the autoimmune pathogenesis of CIU. The receptors for IFN-γ and IL-10 belong to the type 2 cytokine receptors, and their elevated expression is further evidence of Th0 type reaction in CIU, and provides a clue to sorting the components involved in the later phase reactions in CIU[29].

Later phase reaction of allergy has been paid more attention in recent years. Some antihistamines, especially those belonging to the new generation, are claimed with the role of anti-allergic-inflammation, which is related to the action of cytokines at the later phase reactions of allergy[30]. Inhibitory effects of antihistamines upon inflammatory cells, cytokines, chemokines and adhesion molecules are emphasized as important properties of potent antihistamines[31]. We noted that different cytokine profiles were shown by previous studies and different cytokines were targeted by different new antihistamines, which make us speculate that there may be certain regularity in either the expression of cytokine receptors or molecular targets at the later phase reaction of allergic inflammation in CIU. Our data showed that the cytokine receptor expression in CIU was not Th1/Th2 polarized and there were higher levels of *TNFR, IFNγR* and *IL*-*10R* expression as well as higher levels of *CCR3* and *CCR6* expression in CIU patients than those in healthy controls. Chemokine receptors regulate antigen-induced T cell homing[32]. CCR3 is a Th2-associated chemoattractant receptors[33] and CCR6 transcripts were originally reported in lymphocytes and in dendritic cells, but not in monocytes[34],[35]. The interaction between CCR6 and its ligand CCL20 is responsible for the chemoattraction of immature dendritic cells, effector/memory T-cells and B-cells and plays a role at skin and mucosal surfaces under homeostatic and inflammatory conditions as well as in antimicrobial processing[36]. Acosta-Rodriguez recently identified CCR6 as a marker of Th17 cells[37]. Although we did not evaluate the roles of Th17 in our study, the highly expressed CCR6 found by us suggests that Th17 cells might play a role in CIU. The ligand of CCR6 is produced in response to direct TLR and/or cytokine activation and induces recruitment of inflammatory T cells that can amplify the allergic response[38].

Moreover, our data showed markedly decreased levels of mRNA transcripts of *TLR2* and *TLR5* in CIU cases, which indicates a possible linkage between the change of innate immunity and the etiology of CIU although there was not a statistical difference in TLR5 expression between the two groups. TLR2 is essential for the recognition of a variety of PAMPs, including bacterial lipoproteins, peptidoglycan and lipoteichoic acids[39]. TLR2 agonists show high immunomodulatory and adjuvantic capacity. This makes TLR2 agonisation a promising approach for pharmaceutical intervention of allergic disorders, which could be a molecular target in the treatment of CIU as well[40].

Furthermore, through the comparison of the data between the CIU group and the dermographism group, we found that there were higher mean levels of TNFR, IL-10R, and CCR3 in the CIU group than those in the dermographism group with a statistical difference. The clinical significance of this interesting finding remains unclear, which may be associated with their different mechanisms of pathogenesis.

In conclusion, the present study revealed increased mRNA expression of *TNFR, IFN-γR, IL-10R, CCR3* and *CCR6* and decreased *TLR2* mRNA expression in the PBMCs of CIU patients, which could play some roles in the pathogenesis of CIU and need further study.
